# Mesonephric Hyperplasia and Adenocarcinoma of the Cervix: A Rare Evolution, Case Report, and Review of the Literature

**DOI:** 10.3390/reports8040230

**Published:** 2025-11-11

**Authors:** Angel Yordanov, Diana Strateva, Albena Baicheva, Ivan Baichev, Stoyan Kostov, Vasilena Dimitrova

**Affiliations:** 1 Department of Gynaecological Oncology, Medical University Pleven, 5800 Pleven, Bulgaria; strateva_d@abv.bg; 2 Department of General and Clinical Pathology, University Hospital “Dr. Georgi Stranski”, 5800 Pleven, Bulgaria; albena.baycheva@gmail.com (A.B.); ivanbaychev1995@gmail.com (I.B.); 3 Department of Gynaecology, St. Anna University Hospital, Medical University-Varna “Prof. Dr. Paraskev Stoyanov”, 9002 Varna, Bulgaria; drstoqn.kostov@gmail.com; 4 Research Institute, Medical University Pleven, 5800 Pleven, Bulgaria; 5 Faculty of Medicine, Medical University Pleven, 5800 Pleven, Bulgaria

**Keywords:** mesonephric hyperplasia, mesonephric adenocarcinoma, Cervical Cancer, Adenocarcinoma cervix

## Abstract

**Background and Clinical Significance**: Mesonephric adenocarcinoma (MA) of the uterine cervix is an exceptionally uncommon and aggressive cancer that arises from remnants of the mesonephric duct. It was first classified by the World Health Organization (WHO) in the 2020 WHO Classification of Female Genital Tumors as a type of cervical adenocarcinoma, also referred to as Gartner’s duct carcinoma. Due to its rarity, both detection and treatment pose significant challenges, and there is little information on its clinical manifestations and prognosis. Mesonephric hyperplasia (MH) in the uterine cervix is an uncommon condition that is often misdiagnosed as adenocarcinoma. **Case Presentation**: We present the case of a 49-year-old, asymptomatic, perimenopausal woman diagnosed with cervical mesonephric adenocarcinoma following a routine Pap smear, performed by Papanicolaou test, with a III A-B result; however, a cone biopsy revealed stage IB1 mesonephric adenocarcinoma. The patient underwent a radical hysterectomy type C (Querleu–Morrow 2017 classification). The final pathology confirmed stage IB2 of the cancer (2018 classification) according to The International Federation of Gynecology and Obstetrics (FIGO), with previous evidence of mesonephric hyperplasia from a trial abrasion performed three years earlier. **Conclusions**: This case highlights the challenges in recognizing and managing mesonephric hyperplasia and adenocarcinoma of the cervix. Given the uncommon nature of this cancer, clinicians should consider it when treating patients with ambiguous cervical pathology and mesonephric hyperplasia. Optimizing patient outcomes relies on early detection, accurate staging, and radical surgical treatment.

## 1. Introduction and Clinical Significance

Mesonephric adenocarcinoma is a rare histological diagnosis comprising around 1% of cases classified by the 2020 WHO Classification of Female Genital Tumors [1]. It was first described by McGee in 1962 [2]; however, nowadays, fewer than 100 cases have been reported globally, with patients ranging in age from 19 to 85 years [3]. One case has been described involving a 1.5-year-old girl [4].

Growth of this cancer begins with intrauterine development, embryogenesis, and the formation of the female reproductive system from the mesoderm. From this, comes the primitive kidney in utero—the mesonephros. The Mullerian ducts, which grow into the fallopian tubes, uterus, cervix, and upper third of the vagina, run parallel to the mesonephric (Wolffian) ducts, which connect the primitive kidney (mesonephros) to the cloaca. However, as female development advances, the mesonephric ducts regress. Malignant mesonephric tumors are discovered in areas where there are embryonic remnants of Wolffian ducts, known as Gartner’s ducts. This is why mesonephric remnants can be found in the ovarian hilus, the wide ligament, the mesosalpinx, and the lateral wall of the uterus and vagina [5].

This report presents a case of a 49-year-old woman diagnosed with mesonephric adenocarcinoma of the cervix, highlighting a previous diagnosis of mesonephric hyperplasia three years earlier. This report also includes a brief review of the literature to highlight the diagnostic and therapeutic challenges in this case.

## 2. Case Presentation

### 2.1. Medical History

A 49-year-old perimenopausal (last menstruation was 2 months ago) white woman was referred to our Clinic of Gynaecological Oncology with mesonephric adenocarcinoma, FIGO stage IB1 [6]. Her past medical and surgical history was unremarkable, with no previous chronic illnesses or operations. She had one full-term vaginal delivery in 1996 and one elective abortion. Menarche occurred at age 14, with cycles regular from the beginning, occurring every 28 days and lasting 6 days.

Her medical record showed that mesonephric hyperplasia was identified three years prior, following uterine curettage. At that time, she had reported irregular menstruation since the beginning of the year, experiencing heavy menstrual cycles lasting 13 days with blood clots. From the patient’s family history, it was reported that her mother had breast cancer.

### 2.2. Presentation and Work-Up

During a gynecological preventative exam, a Pap smear performed by Papanicolaou test was taken, showing a III A-B result. Colposcopy was performed with normal findings. However, the patient remained asymptomatic and had no history of abnormal uterine bleeding or pelvic pain.

A cone biopsy was performed, revealing stage IB1 mesonephric adenocarcinoma with negative macroscopic margins (RO) [7].

On preoperative physical examination, the patient had an ECOG performance status of 0. The external genitalia were non-pathological, and speculum examination revealed a normal vaginal canal with a cervix appearing normal post-cone biopsy and without visible macroscopic lesions. Bimanual palpation showed a normal, mobile cervix, a uterus appropriate for age, and adnexa without abnormalities. Per rectal examination confirmed free parametria on both sides.

No suspicious findings were revealed during the physical exam.

**Figure 1:**
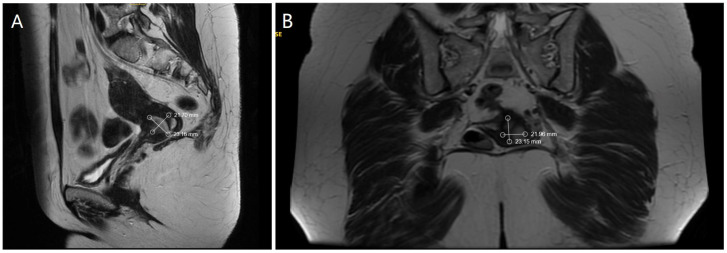
(**A**). MRI—Sagittal section of the tumor formation (**B**). MRI—Coronal section of the tumor formation.

Ultrasound examination of the abdomen and pelvis revealed no abnormal findings, and lung radiography was normal. Preoperative magnetic resonance imaging (MRI) identified an infiltrative tumor measuring 23.69 × 21.70 mm, as demonstrated in Figure 1A,B.

### 2.3. Surgical Treatment

**Figure 2:**
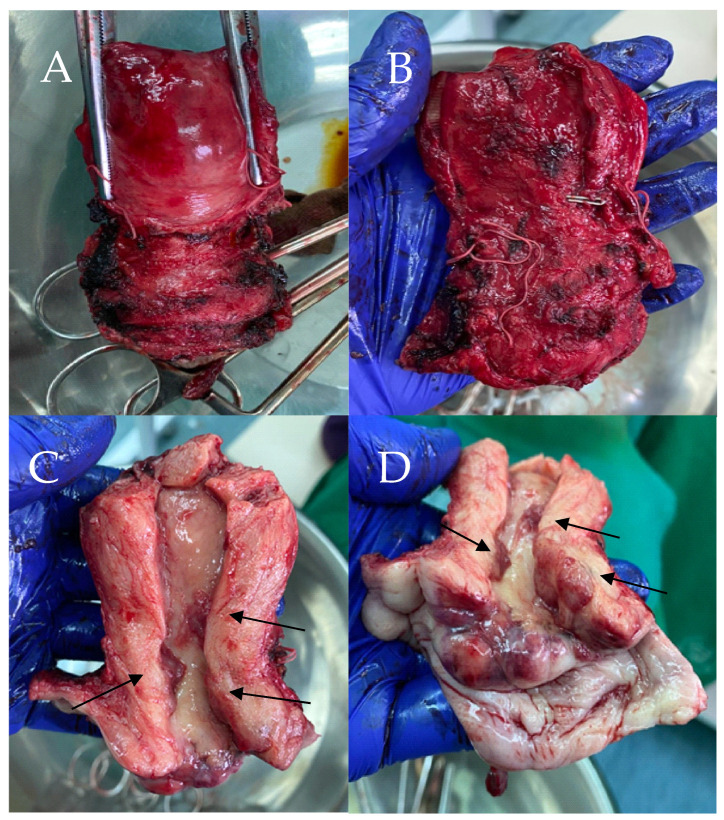
Macroscopic view of specimen (**A**). Anterior view of specimen (**B**). Posterior view of specimen (**C**). Cavity of specimen with the papillary lesions (**D**). Specimen with the papillary lesions.

The patient subsequently underwent a radical hysterectomy type C according to the 2017 Querleu–Morrow classification [8]. During surgery, the uterus was in an anteverted flexed (AVF) position, and was normal in shape and size; however, dense adhesions were present from the rectum to the posterior uterine wall. After removal and sectioning, the cervix showed multifocal papillary lesions, marked in Figure 2C,D.

Final pathology findings confirmed FIGO stage IB2 [6] mesonephric adenocarcinoma, likely due to increased tumor dimensions observed postoperatively. The difference between the FIGO staging determined by the cone biopsy and the final pathological findings after surgery is attributable to the high location of the tumor within the cervix, as well as the presence of multifocal lesions, which became evident only after the conization.

### 2.4. Pathological Findings

**Figure 3:**
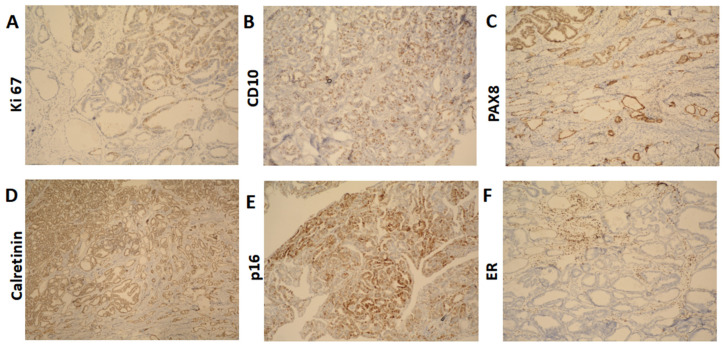
Immunohistochemical results (**A**). Ki 67 ×100 (**B**). CD10 ×100 (**C**). PAX8 ×100 (**D**). Calretinin ×100 (**E**). p16 ×100 (**F**). ER ×100.

**Figure 4:**
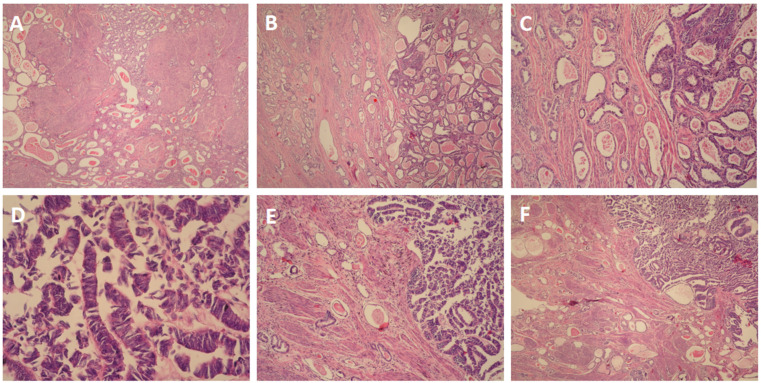
(**A**). H&E ×4 (**B**). H&E ×10 (**C**). H&E ×100 (**D**). H&E ×400 (**E**). H&E ×100 (**F**). H&E ×10.

The cervix was infiltrated by a tumor, represented by ducts, fibrous papillae in dilated slits lined by cubic epithelium with scanty cytoplasm, pronounced atypia, and proliferative activity Ki 67 on about 25% of the tissue (Figure 3A). The tumor infiltrated the entire thickness of the cervix, including the outer posterior edge, infiltrated the stroma in the isthmus, and formed an exophytic formation in the cervical canal. The tumor dimensions were as follows: a depth of about 3 cm, and a horizontal size of 4 cm. Representative sections stained with hematoxylin and eosin (H&E) highlight these histological features (Figure 4A–F). The 18 lymph nodes excised exhibited follicular hyperplasia. Tumor invasion was not described in the adnexa and parametrial tissue.

The immunohistochemical findings were as follows: CD10: positive (+) (Figure 4B); PAX8: diffuse positive (+) (Figure 4C); Calretinin: focal positive (+) (Figure 4D); p16: focal positive (+) (Figure 4E), indicating it is not HPV-driven, which aligns with the characteristics of mesonephric adenocarcinoma; ER: negative (−) (Figure 4F); TTF1: negative (−); CEA: negative (−).

### 2.5. Adjuvant Therapy

Following surgery, the patient received adjuvant radiotherapy consisting of intravaginal brachytherapy with a total dose of 3.2 Gy, followed by external beam radiotherapy (EBRT) to the lesser pelvis and cervix using the Intensity-Modulated Radiation Therapy (IMRT) technique, with a daily dose of 1.8 Gy up to a total dose of 50.4 Gy. The treatment was well tolerated, resulting in improved local tumor control and no change in the patient’s general condition.

### 2.6. Follow-Up

The patient was discharged in good general condition, afebrile, with restored micturition and defecation. She has been under regular follow-up for one year after surgery. A positron emission tomography/computed tomography (PET/CT) was performed at the one-year mark, showing no pathological findings or evidence of disease recurrence.

## 3. Discussion

Mesonephric adenocarcinoma (MA) of the cervix is a rare and aggressive malignancy arising from remnants of the mesonephric (Wolffian) duct. Due to its rarity, it is often misdiagnosed or detected late, making early identification crucial for patient prognosis. Our case is unique because it presents a documented instance of mesonephric hyperplasia three years before the development of carcinoma, providing rare insight into the natural history of mesonephric neoplasia.

One of the key challenges in diagnosing mesonephric adenocarcinoma is its histological overlap with other types of cervical adenocarcinomas, particularly clear cell carcinoma and endometrioid adenocarcinoma. However, our case was distinguished by immunohistochemical markers characteristic of MA, including CD10 and Calretinin positivity, with the absence of HPV-driven markers such as p16 [9]. The diagnosis was further complicated by the asymptomatic nature of the patient and the absence of colposcopic atypism, highlighting the importance of vigilant screening and histopathological assessment.

Mesonephric adenocarcinoma (MA) of the uterine cervix is a rare and diagnostically challenging neoplasm that arises from remnants of the mesonephric (Wolffian) duct system. Increasing evidence from the literature and our case study supports the hypothesis that mesonephric hyperplasia may serve as a precursor lesion to mesonephric adenocarcinoma, with the florid [10], diffuse [11], and lobular [11] subtypes in particular serving as precursor lesions.

Up to 22% of adult female cervixes have residues of the mesonephric ducts, which are often found deep within the lateral walls of the cervix. These remnants may give rise to mesonephric hyperplasia and, in rare cases, progress to malignant mesonephric tumors [12]. This underscores the importance of thorough histopathological evaluation when mesonephric hyperplasia is encountered in cervical specimens, as well as the need for close clinical follow-up. In our case, the patient initially presented with mesonephric hyperplasia three years prior to the diagnosis of mesonephric adenocarcinoma, supporting the hypothesis that prolonged hyperplastic changes may undergo malignant transformation over time. The relationship between hyperplasia and carcinomas is not clearly established; due to the disease’s etiology, careful monitoring is recommended, although increasing evidence suggests a possible precursor role.

In order to understand the origin of this tumor, we recapitulated both current and previous cases of mesonephric hyperplasia, detected at different stages, including our case. We performed a literature search in English using PubMed and Google Scholar, with the following terms: “Mesonephric hyperplasia, Mesonephric adenocarcinoma, Cervical Cancer, Adenocarcinoma cervix”. In total, around 40 cases met the criteria, and are summarized in Table 1.

To the best of our knowledge, Table 1 integrates a review of over 40 published cases from 1987 to 2022—including our case—revealing a consistent pattern of MH preceding or coexisting with MA. In seminal studies by Clement et al. (1995) and Silver et al. (2001), florid, diffuse, or lobular MH was identified in the majority of tumors—7 out of 8 and 10 out of 11 cases, respectively [18,19]. These findings support the concept of a biological continuum with MH, particularly when atypical or proliferative, representing a precursor lesion capable of progressing to carcinoma.

The stepwise progression from MH to MA is further supported by reports in the literature. Cases by Puljiz et al. (2016), Fukunaga et al. (2018), and Abdul-Ghafar et al. (2013) illustrate continuity between florid or lobular hyperplasia and invasive carcinoma [12,21,25]. In the study by Secosan et al. (2022), MH was detected five years before the diagnosis of MA; in our present case, the interval was three years [26]. These examples reinforce the need for long-term follow-up in patients with hyperplastic mesonephric lesions.

Histologic evaluation is critical, especially given the heterogeneity of mesonephric proliferations. While many cases of MH remain benign, others—particularly those with florid, diffuse, or atypical architecture—are more likely to undergo malignant transformation. The case studies by Ferry et al. (1990) and Bagué et al. (2004) included both benign MH and aggressive variants, such as malignant mixed mesonephric tumors (MMMTs), further highlighting this spectrum of behavior [16,20].

The variability in clinical course is particularly evident in MMMTs, which contain both glandular (epithelial) and sarcomatous components. These tumors, described by Bagué et al., Bloch et al. (1988), and Meguro et al. (2013), tend to exhibit aggressive clinical behavior and often present at advanced stages [14,20,22]. Heterologous elements such as osteosarcoma and cartilage were found to be associated with recurrence and metastasis, including mediastinal and bone spread. Conversely, many patients with early-stage pure MA had favorable outcomes following surgical treatment, as shown in cases by Silver et al. and others.

Analysis of the compiled cases also reveals broad demographic distribution, with affected patients ranging in age from their 20s to mid-60s. Vaginal bleeding was the most common symptom, yet some cases were detected incidentally. Treatment protocols were relatively consistent, with most patients undergoing hysterectomy, often combined with bilateral salpingo-oophorectomy and lymphadenectomy. Tumor sizes varied widely (1.8 cm to over 8 cm), though most cases were diagnosed at FIGO stage I.

Outcomes were generally favorable for early-stage MA. Several patients remained disease-free for up to 13 years post-treatment. However, recurrence and metastasis were not uncommon in cases with advanced disease, mixed histology, or sarcomatous components. Bagué et al. reported a patient with stage IV MMMT and bone metastases, while Silver et al. documented deaths from recurrent or metastatic disease, underscoring the importance of early detection.

Mesonephric hyperplasia (MH), especially in its florid, diffuse, or atypical forms, appears to be a plausible precursor to mesonephric adenocarcinoma (MA), as supported by both our case and a broad review of the literature. Accurate histopathological and immunohistochemical evaluation is essential for distinguishing MA from other cervical malignancies, particularly given its frequent asymptomatic presentation and diagnostic overlap. Pure mesonephric adenocarcinomas tend to present at early stages and have favorable outcomes with surgical management alone, whereas malignant mixed mesonephric tumors (MMMTs), often containing sarcomatous components, exhibit more aggressive clinical behavior and poorer prognoses. Early detection and appropriate staging are critical for improving outcomes.

## 4. Conclusions

Mesonephric adenocarcinoma of the cervix is an exceedingly rare and diagnostically challenging malignancy that often mimics other cervical adenocarcinomas. This case underscores the critical role of mesonephric hyperplasia as a potential precursor lesion, with our patient developing MA three years after an initial diagnosis of MH.

A thorough review of the literature further reinforces the hypothesis that florid, diffuse, or atypical MH may represent a biologically active precursor to MA, with documented progression in several cases. While pure MA often presents at an early stage and responds well to surgical treatment, malignant mixed mesonephric tumors can exhibit aggressive behavior and a higher risk of recurrence.

Early identification, accurate histopathologic evaluation, and appropriate staging remain fundamental to improving prognosis. Given the potential for delayed malignant transformation, clinicians should maintain a high index of suspicion when encountering mesonephric remnants or hyperplasia and ensure long-term surveillance. Further molecular and clinical studies are warranted to clarify the pathogenesis and guide standardized management protocols for these rare tumors.

## Figures and Tables

**Table 1:** Summary of mesonephric hyperplasia of the cervix reported in the literature, including the present case.

№	Age	Type MH	Years to CA	Diagno-Sis	Treat-Ment	TU Size	N Stage	FIGO 2018	Outcome
Valente et al. (1987) [13]	58	florid	N/A	MA arising in florid MH	RAH, BSO, PLND	N/A	N/A	IB	34 mo Local recurrence
Bloch et al. (1988) [14]	65	Atypical MH	N/A	Osteosarcoma associated with mesonephric rests	N/A	3 cm	N/A	IB	1 yr 11 mo NED
Ferry et al. (1990) [15]—Case 1	48	Diffuse	N/A	MA arising in diffuse MH	Biopsy	N/A	N/A	N/A	9 yr Local recurrence
Ferry et al. [15]—Case 2	58	Diffuse	N/A	A arising in diffuse MH	Hysterectomy	N/A	N/A	IB	10 mo Local recurrence, 2 yr died
Ferry et al. [15]—Case 3	55	Lobular or Diffuse	N/A	MA	Hysterectomy	N/A	N/A	N/A	N/A
Ferry et al. [15]—Case 4	36	Lobular or Diffuse	N/A	MA	Hysterectomy	N/A	N/A	IB	Local recurrence 6 yr, died 7 yr
Lang et al. (1990) [16]	46	N/A	N/A	N/A	Uter	N/A	N/A	N/A	10 mo NED
	55	N/A	N/A	N/A	Uter	N/A	N/A	IB	N/A
Stewart et al. (1993) [17]	37	diffuse	N/A	MA arising in diffuse MH	RH, PLND	N/A	N/A	IB1	10 yr NED
Clement et al. (1995)—Case 1 [18]	34	Florid/atypical	N/A	MA + sarcomatoid	Hysterectomy	N/A	Micromets	IB	3 yrs—NED
Clement et al.—Case 2 [18]	~40 s	Florid/atypical	N/A	MA + osteosarcoma	Hysterectomy	N/A	Micromets	IB	13 yrs—alive w/tumor
Clement et al.—Case 3 [18]	~40 s	Present	N/A	MA + spindle cell	Hysterectomy	N/A	N/A	IB	2 yrs—NED
Clement et al.—Case 4 [18]	~50 s	Present	N/A	MA + chondroid focus	Hysterectomy	N/A	N/A	IB	1 yr—NED (post-chemo)
Silver et al. (2001)—Case 1 [19]	35	Present	N/A	MA	Hysterectomy	N/A	N/A	IB	Alive—NED (4.8 yrs avg)
Silver et al.—Case 2 [19]	52	Present	N/A	MA	Hysterectomy	N/A	N/A	IB	Alive—no recurrence (4.8 yrs avg)
Silver et al.—Case 3 [19]	N/A	Present	N/A	MA	Hysterectomy	N/A	N/A	IIB	Died at 3.2 yrs—recurrence
Silver et al.—Case 4 [19]	N/A	Present	N/A	MA	Hysterectomy	N/A	N/A	IVB	Died at 0.8 yrs—Local recurrence
Silver et al.—Case 5 [19]	N/A	Present	N/A	MA	Hysterectomy	N/A	N/A	IB	Died at 6.2 yrs—Distant recurrence
Bagué et al. (2004)—Case 1 [20]	54	Present	N/A	MA	RAH + BSO	2 cm	N0	IB	3 yrs—NED
Bagué et al.—Case 2 [20]	24	Present	N/A	MA	RAH + BSO + LND	6 cm	N0	II	11.5 yrs—NED
Fukunaga et al. (2018) [21]	46	Lobular	After surgery	MA w/lobular MH	TAH, BSO, PLND, O	4 cm	N0	IB	4 mo NED
Meguro et al. (2013) [22]	63	Diffuse	After surgery	MA with sarcomatous component arising in MH	RAH, BSO, PLND	1.8 cm	N0	IIA	7 mo- Local recurrence; after 3 mo-NED
Menon (2013) [23]	65	Diffuse	N/A	MA of endometrioid type with squamous morules in association with diffuse MH	TAH, BSO, PLND	N/A	N/A	IB1	6 mo NED
Abdul-Ghafar et al. (2013) [12]	48	florid	After surgery	MA with florid MH	Vaginal hysterectomy	3.5 × 2.5 × 2.5 cm	N0	IB1	2 yr NED
Tekin et al. (2015) [24]	64	N/A	N/A	MA	TAH, BSO, PLND, PALND	5 cm	N0	IB2	N/A
Puljiz et al. (2016) [25]	57	Lobular	After surgery	MA w/lobular MH	RH + BSO + LND	5.4 × 4 cm	N0	IB3	3 yrs—NED
Nili et al. (2021) [26]	46	Florid	Underdiagnosed	MA	SH + BS	4.5 × 4 × 3 cm	Nx	IB3	9 mo—Local and distant recurrence
Secosan et al. (2022) [27]	29	–	5 yrs	MA w/atypical MH	RVT + LPLND	2.2 × 1.6 × 1.6 cm	N0	IB2	3 yrs—NED
Present case	49	–	3 yrs	MA	RH + BSO + LND	3 × 4 cm	N0	IB2	1 yr—NED

N/A—Not applicable; MH—mesonephric hyperplasia; MA—mesonephric adenocarcinoma; RAH—radical abdominal hysterectomy; BSO—bilateral salpingo-oophorectomy; PLND—pelvic lymph node dissection; PALND—para-aortic lymph node dissection; TAH—total abdominal hysterectomy; RH—radical hysterectomy; LND—lymph node dissection; SH—simple hysterectomy; BS—bilateral salpingectomy; mo—month(s); yr—year(s); NED—no evidence of disease.

## Data Availability

The original contributions presented in this study are included in the article. Further inquiries can be directed to the corresponding authors.
